# Estrogen reprograms the activity of neutrophils to foster protumoral microenvironment during mammary involution

**DOI:** 10.1038/srep46485

**Published:** 2017-04-21

**Authors:** Hwa Hwa Chung, Yu Zuan Or, Smeeta Shrestha, Jia Tong Loh, Chew Leng Lim, Zoe Ong, Amanda Rui En Woo, I-Hsin Su, Valerie C. L. Lin

**Affiliations:** 1 School of Biological Sciences, Nanyang Technological University, 60 Nanyang Drive, Singapore 637551, Singapore

## Abstract

Epidemiological studies have indicated increased risk for breast cancer within 10 years of childbirth. Acute inflammation during mammary involution has been suggested to promote this parity-associated breast cancer. We report here that estrogen exacerbates mammary inflammation during involution. Microarray analysis shows that estrogen induces an extensive proinflammatory gene signature in the involuting mammary tissue. This is associated with estrogen-induced neutrophil infiltration. Furthermore, estrogen induces the expression of protumoral cytokines/chemokines, COX-2 and tissue-remodeling enzymes in isolated mammary neutrophils and systemic neutrophil depletion abolished estrogen-induced expression of these genes in mammary tissue. More interestingly, neutrophil depletion diminished estrogen-induced growth of ERα-negative mammary tumor 4T1 in Balb/c mice. These findings highlight a novel aspect of estrogen action that reprograms the activity of neutrophils to create a pro-tumoral microenvironment during mammary involution. This effect on the microenvironment would conceivably aggravate its known neoplastic effect on mammary epithelial cells.

Although full-term pregnancy in young women reduces the lifetime risk of breast cancer, it has long been established that there is a transient increase of breast cancer risk within 10 years following childbirth^1^. The risk of such parity-associated breast cancer(PABC) increases with age at delivery. As more women are delaying their child-bearing, the number of PABC cases is set to rise. It is thus pivotal to understand the etiology of PABC. Like all types of cancers, PABC is a complex disease and its etiology is likely multifaceted involving both endogenous and environmental factors. The breasts undergo mammary involution after weaning, characterized by acute inflammation, programmed cell death and tissue remodeling^2^. Studies have suggested that mammary inflammation and tissue remodeling during involution promote mammary malignant transformation through collagen and cyclooxygenase-2(COX-2)^3^,^4^, indicating that COX-2 inhibitors are useful agents for the prevention of PABC.

Breast cancer is also an endocrine disease. While estrogen is required for mammary ductal growth during normal development, estrogen exposure is also a well-known risk factor for breast cancer^5^. Over 50% of all breast cancers are estrogen-dependent at the time of diagnosis^6^. The canonical model for the pro-tumoral action of estrogen involves primarily estrogen receptor α(ERα) that acts on both genomic and non-genomic pathways^7^. Estrogen-activated ERα up-regulates proto-oncogenes and down-regulates tumor suppressor genes by interacting directly with the estrogen response element of its target genes. Activated ERα also interacts with signaling cascades such as PI3K-ILK-AKT and Src-ERK to transmit growth signals^8^. There has also been evidence that estrogen promotes tumor development through the tumor microenvironment. This appears to involve the immunomodulatory function of estrogen. It has been reported that estrogen could promote protumoral microenvironment by inducing the recruitment of bone marrow-derived cells^9^ and by inducing macrophage infiltration and release of CCL2 and CCL5^10^.

It has been increasingly recognized that tumor-associated neutrophils play pivotal roles in tumor biology. Fridlender *et al*.^11^ first suggested the existence of N1(antitumoral) and N2(protumoral) neutrophils, analogous to the concept of tumor-associated M1 and M2 macrophages^12^. The N2 neutrophils promote malignancy by releasing protumoral cytokines and chemokines, matrix-degrading proteases, mediators of angiogenesis^13^. In a wound-healing tumor model, neutrophils recruited to the wound quickly migrate to the nearby tumor cells and drove their proliferation^14^. Neutrophils were also found to conspire with IL-17-producing γδ T cells to promote cancer metastasis^15^. Interestingly, neutrophils are the first leucocytes recruited to mammary tissue during involution^16^. However, the involvement of neutrophils in tumorigenesis during mammary involution has not been studied.

Although the molecular and cellular effects of estrogen on mammary development and breast cancer have been studied extensively, little is known about how estrogen influences mammary involution. So far, it has been shown that injection of estrogen in dairy cows during the drying off period inhibits milk synthesis and secretion^17^. In light of the significance of mammary inflammation during involution in tumor development and the involvement of estrogen in tumor microenvironment, we investigated the effect of estrogen on mammary inflammation during involution and addressed the impact of this immunomodulatory effect on mammary tumor development. The study shows that estrogen heightens mammary inflammation and induces neutrophils infiltration. More importantly, estrogen elicits a marked reprogramming of neutrophil activity to foster a protumoral tissue environment during mammary involution. Consequently, neutrophil depletion abolished estrogen-induced mammary tumor growth during mammary involution. We propose that minimizing estrogen exposure during mammary involution is important to reduce the incidence of PABC. This study also highlights pivotal roles of estrogen in modulating neutrophil activity that could have significant implications in ERα-negative breast cancer.

## Results

### Estrogen induces striking inflammatory gene signature

**Figure 1:**
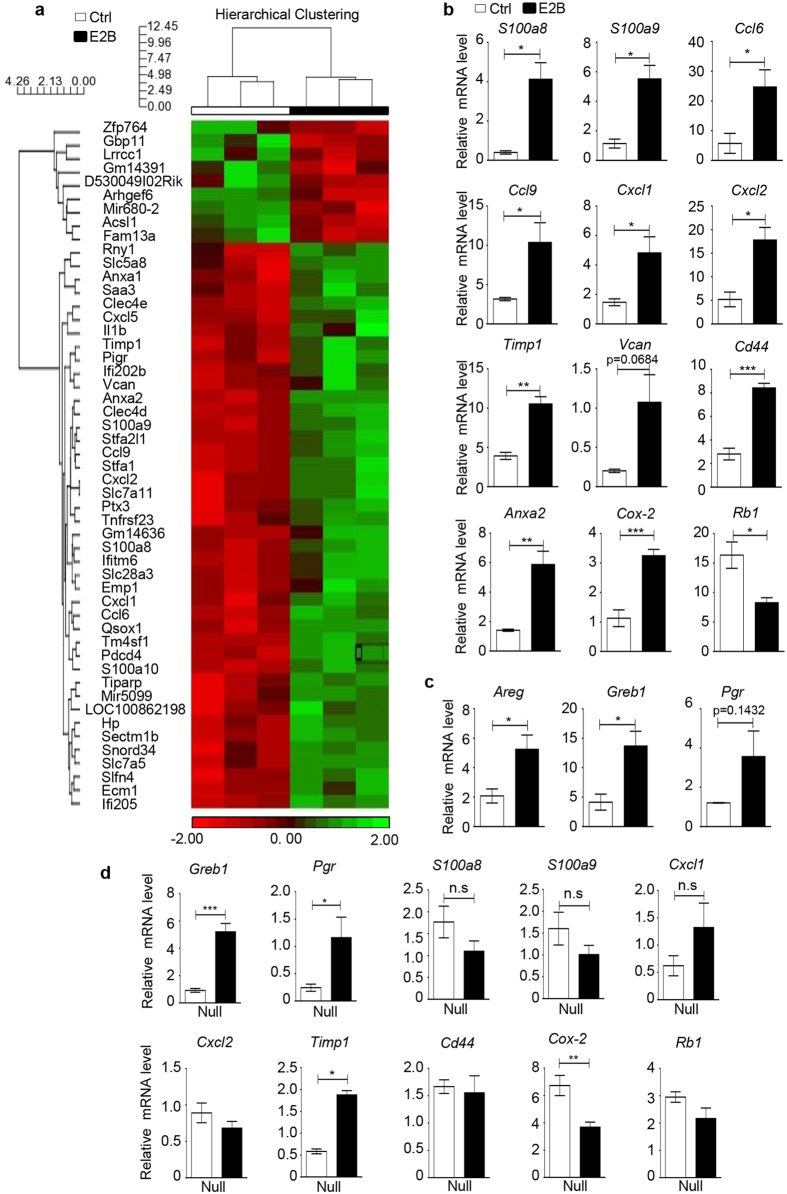
Microarray analysis of the effect of estrogen on global gene expression in mammary tissue during involution. Mammary glands of mice at 48 h involution were treated with Ctrl(n = 3) or E2B in sesame oil(n = 3) for 24 h. Total mammary RNA was analyzed for E2B-induced gene expression using Affymetrix Mouse Gene 2.0 ST array.(**a**) Heat map of E2B-regulated genes with fold change ≥2.0 and p value < 0.05.(**b**) RT-PCR validation of a panel of 12 E2B regulated genes.(**c**) Known estrogen target genes *Areg, Greb1* and *Pgr* were also upregulated after 24 h E2B treatment in involuting mice.(**d**) Estrogen-induced immune response gene expression except for *Timp1* in involuting mammary does not occurs in the mammary tissue of nulliparous mice. Ctrl, n = 3; E2B, n = 3. All data are presented as Mean ± SEM. White and black bars represent Ctrl and E2B treatment, respectively. Statistical significance was evaluated by unpaired two-tailed Student’s t-tests; *p < 0.05, **p < 0.01, ***p < 0.001.

To gain a whole genomic understanding of the the molecular effect of estrogen on the mammary gland during involution, we conducted microarray analysis of estrogen-regulated gene expression using Affymetrix Mouse GeneChip 2.0 ST array. After 24 h treatment, 51 genes were significantly(p < 0.05) regulated by E2B with fold change of greater than 2(Fig. 1a), and 239 genes with fold change of greater than 1.5(Supplementary Table 2). Ingenuity Pathway Analysis(IPA) and GeneGo MetaCore analysis revealed that estrogen-induced gene expression changes were significantly enriched in pathways related to inflammatory responses and cell adhesion(see top 15 pathways in Supplementary Fig. 1a and b). In particular, IL-17A and IL-17F pathways, which have been linked to neutrophil expansion and polarization in mice bearing mammary tumors^15^ are significantly enriched in both analyses.

The microarray data were validated by real-time PCR(RT-PCR) analysis of a panel of 10 genes that are involved in immune function or cell adhesion(*S100a8, S100a9, Ccl6, Ccl9, Cxcl1, Cxcl2, Timp1, Vcan, Cd44* and *Anxa2*)(Fig. 1b). The PCR analysis also verified that estrogen up-regulated the expression of the gene encoding COX-2(*Ptgs2*) and down-regulated the expression of tumor suppressor gene *Rb1*(Fig. 1b). Although the known estrogen target genes Growth Regulation by Estrogen in Breast cancer 1(*Greb1*), Amphiregulin(*Areg*), and Progesterone Receptor(*Pgr*) were not present in the list of estrogen-regulated genes by microarray analysis, these genes were found to be significantly up-regulated by RT-PCR analysis(Fig. 1c).

Since this comprehensive induction of immune response genes by estrogen in mammary gland has not been reported before, we wondered if the estrogen response is unique to the mammary gland during involution. The effect of estrogen on the expression of a panel of genes was tested in the mammary gland of age-match nulliparous mice. We first verified that *bona fide* estrogen target genes *Greb1* and *Pgr* are significantly up-regulated in these age-match nulliparous mice(Fig 1d). We then tested 8 genes(*S100a8, S100a9, Cxcl1, Cxcl2, Cd44, Rb1, Timp1 and Cox-2*) that are regulated by estrogen in involuting mammary gland. Estrogen had no effect on the expression of *S100a8, S100a9, Cxcl1, Cxcl2, Cd44* and *Rb1* in nulliparous mice. Cox-2 is down-regulated in nulliparous mice as opposed to the upregulation in involuting mice. The only gene that is similarly regulated in both nulliparous and involuting mammary tissue is *Timp1*. The observations indicate that estrogen action on the mammary gland is dependent on the tissue microenvironment.

### Estrogen-induced inflammatory gene signature occurs primarily in leucocytes

**Figure 2:**
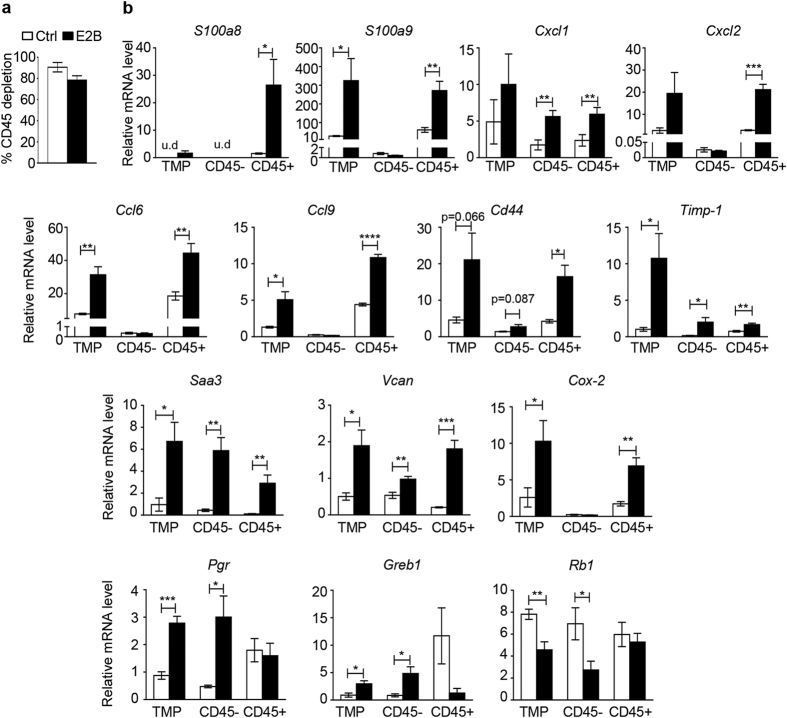
Estrogen-induced expression of immune response genes during mammary involution occurs mainly in leucocytes. Mice at 24 h post-weaning were treated with Ctrl or E2B in sesame oil for 48 h. Mammary tissues were digested to obtain single cell suspension. CD45-positive leucocytes were separated from CD45− cells using Dynabeads^®^.(**a**) CD45-positive cell depletion efficiency was assessed using FACS.(**b**) RT-PCR analysis of the expression of various genes regulated by estrogen in total mammary population(TMP), CD45-depleted(CD45−) and CD45-positive(CD45+) cells. Results are presented as Mean ± SEM. n = 4 mice per group. For all graphs, white and black bars represent Ctrl- and E2B-treated group, respectively. Statistical significance was evaluated by unpaired two-tailed Student’s t-tests; *p < 0.05, **p < 0.01, ***p < 0.001. u.d., undetected.

Acute mammary inflammation during involution is associated with infiltration of immune cells. Since estrogen receptors are expressed in both lymphoid and myeloid cells^18^, we asked if estrogen-induced immune response gene signature occurred in the leucocytes of the mammary gland. Mammary leucocytes were isolated using biotin-labelled antibody against leucocyte common antigen CD45 coupled to Dynabeads^®^ Streptavidin with a depletion efficiency of 75–100%(Fig. 2a). Samples incubated with beads without CD45 antibody represent total mammary cells population(TMP). As expected, analysis of TMP confirmed the microarray data. Of the 11 genes tested, 6 genes(*S100a8, S100a9, Ccl6, Ccl9, Cxcl2* and *Cd44*) were induced by E2B only in CD45+ cells. *Cxcl1* and *Saa3*, which are also involved in acute inflammatory response, were significantly induced by E2B both in CD45+ and CD45− populations(Fig. 2b). Cell adhesion and tissue remodeling genes *Vcan* and *Timp1* were also induced in both CD45+ and CD45− populations. On the other hand, the well-established estrogen target genes *Pgr, Greb1* and tumor suppressor gene *Rb1* were induced only in CD45− cells(Fig. 2b), which are mostly mammary epithelial cells. Hence, estrogen exerts distinct gene regulatory effects on different cell populations of the mammary tissue and the estrogen-induced inflammatory gene signature occurs mainly in immune cells.

### Estrogen promotes infiltration of neutrophils during mammary involution

**Figure 3:**
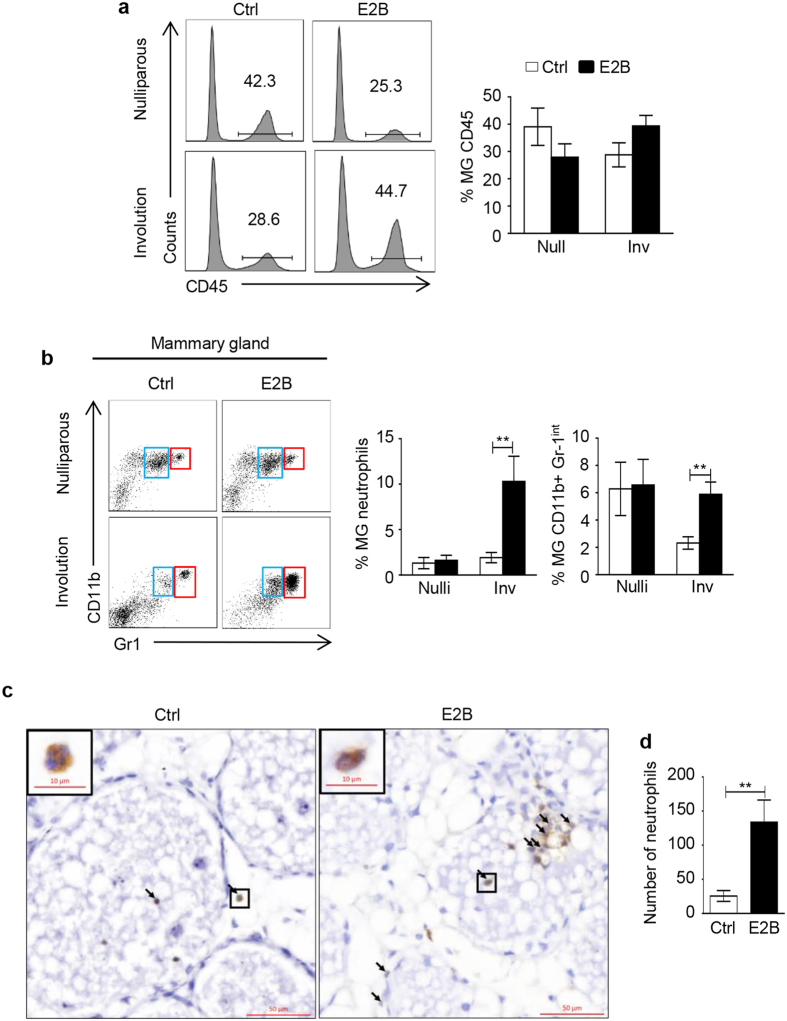
Estrogen promotes neutrophil recruitment during mammary tissue involution. Mice at 24 h post-weaning or age-matched nulliparous mice were treated with Ctrl or E2B in sesame oil for 48 h. Mammary tissues were digested by collagenase to obtain single cell suspension. Mammary cells and blood samples were stained with various cell surface markers.(**a**) E2B treatment did not affect total CD45^+^ cell infiltration in involuting or nulliparous mammary tissue. Representative histograms were shown for CD45^+^ in Ctrl- and E2B-treated nulliparous and involuting mammary glands.(**b**) Estrogen treatment induced significant neutrophils and myeloid-derived monocytic cells infiltration to involuting but not to nulliparous mammary tissue.(Left) Representative dot plots of neutrophils(CD45^+^CD11b^+^Gr-1^hi^)(red boxes) and myeloid-derived monocytic cells(CD45^+^CD11b^+^ Gr1^int^)(blue boxes) from mammary glands CD45+ cell population.(Right) Bar graphs show the percentages of neutrophils and myeloid-derived monocytic cells in total cell population. For the data on mammary tissue in(**a**,**b**) from nulliparous mice, Ctrl n = 10, E2B n = 9; for the data of mammary tissue in(**a**,**b**) from mice undergoing involution, Ctrl n = 13, E2B n = 13.(**c**) Gr-1 IHC of Ctrl- or E2B-treated involuting mammary tissue. Scale bar = 50 μm.(**d**) Total number of infiltrated neutrophils in mammary tissue were counted in an area of 20 mm^2^ for each sample. n = 9 per group. White and black bars represent Ctrl and E2B treatment, respectively. Statistical significance was evaluated by unpaired two-tailed Student’s t-tests, **p < 0.01.

Since estrogen-induced chemokines and cytokines such as S100A8, S100A9, CXCL1 and CXCL2 are among the most potent chemoattractants^19^, we tested if E2B-induced expression of these genes is associated with increased infiltration of immune cells. FACS analysis showed that E2B treatment had no effect on the recruitment of total leucocytes, defined by the expression of CD45 antigen(Fig. 3a). However, E2B significantly modified the profiles of the infiltrated leucocytes in the involuting mammary gland by increasing the infiltration of CD11b^+^ Gr-1^hi^ neutrophils(1.92 ± 0.56 in control samples vs 10.92 ± 2.92 in E2B-treated samples, p < 0.01) and CD11b^+^ Gr-1^int^ myeloid-derived monocytic cells(2.32 ± 0.46 in control samples vs 5.79 ± 0.97 in E2B-treated samples, p < 0.01)(Fig. 3b). On the other hand, E2B had no effect on the recruitment of macrophages(both F4/80^+^ CD11c^−^ and F4/80^+^ CD11c^+^ macrophages)(Supplementary Fig. 2a).

The data also indicated that E2B reduced CD4^+^ and CD8^+^ T cells in mammary tissue by 5- and 4-fold, respectively(Supplementary Fig. 2a). Due to large variations, the reduction was not statistically significant(p > 0.05). There was no difference in the percentage of γδ T cells and dendritic cells between E2B-treated and control group.

E2B-induced neutrophil infiltration in mammary tissue was verified by immunohistochemical staining using antibody against Gr-1. Although the Gr-1 antibody is known to recognize both Ly6C and Ly6G and may stain for both monocytes and neutrophils^20^, we used an additional criterion for accurate neutrophil quantification, which is the presence of discernible banded or segmented nucleus characteristic of neutrophils. Consistent with the FACS data, the number of infiltrated neutrophils in the E2B-treated group increased by ~8-fold compared to the vehicle-treated controls(Fig. 3c,d).

On the other hand, E2B had no effect on mammary neutrophil recruitment in age-matched nulliparous mice(Fig. 3b). The differential effects of estrogen between nulliparous and mice undergoing mammary inflammation suggest that the inflammatory microenvironment of the involuting mammary gland exerts a priming effect on estrogen regulation of mammary inflammation. However, E2B did induce a similar decrease in mammary CD4^+^ and CD8^+^ T cell populations in nulliparous mammary tissue as that in involuting mammary glands(Supplementary Fig. 2a), which is consistent with reports that estrogen inhibits T cell lymphopoiesis in the thymus^21^.

### Estrogen targets neutrophils specifically to induce inflammatory gene signature

**Figure 4:**
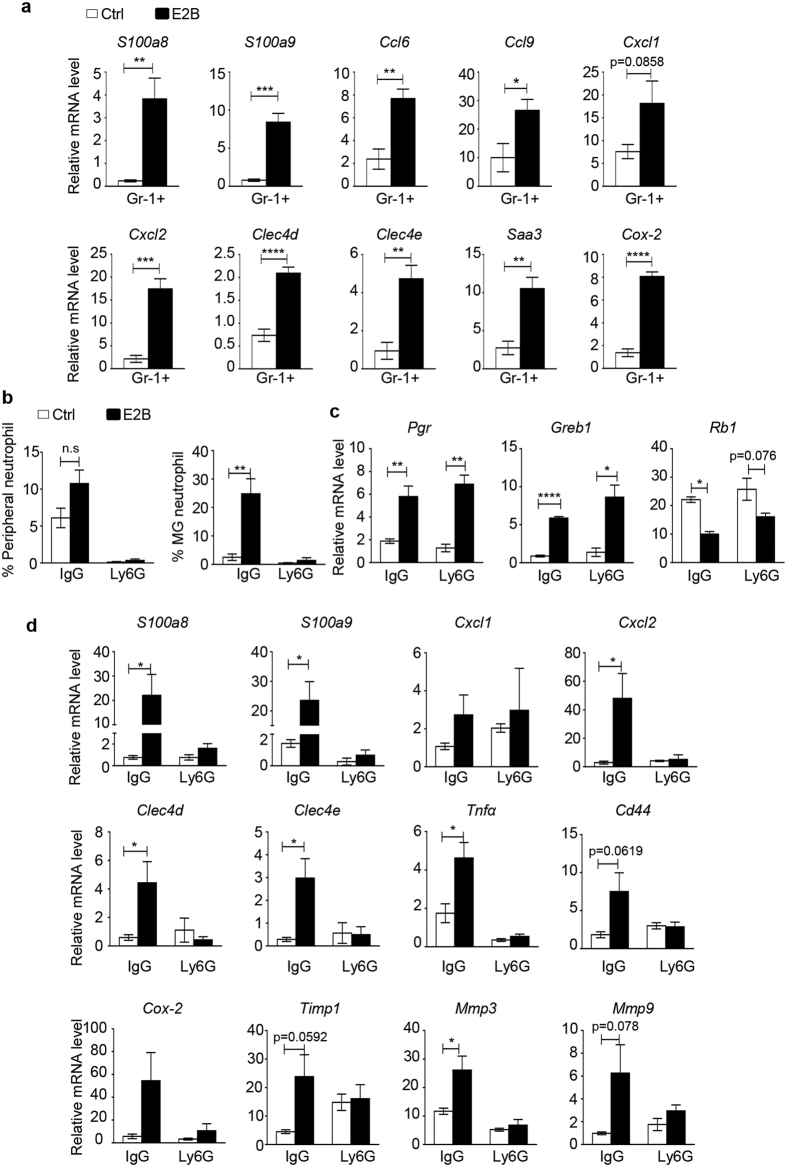
Estrogen targets neutrophils specifically to induce inflammatory gene signature. (**a**) Mice at 24 h post-weaning were treated with Ctrl(n = 4) or E2B(n = 4) in sesame oil for 48 h. Neutrophils were isolated from mammary tissues using biotinylated anti-Gr-1 antibody coupled to Streptavidin Dynabeads^®^ and total RNA from neutrophils were analyzed for gene expression by RT-PCR. The mRNA levels relative to 36B4 are expressed as Mean ± SEM.(**b**,**c**,**d**) Depletion of neutrophils abolishes estrogen-induced gene expression in involuting mammary glands. Mice at 24 h post-weaning were administered with isotype control IgG or Ly6G antibody followed by treatment with Ctrl or E2B in sesame oil for 48 h.(**b**) Ly6G antibody significantly reduced the percentages of neutrophils in blood(left) and mammary gland(right) by FACS analysis.(**c**,**d**) Neutrophil depletion abrogated E2B-induced expression of immune response genes but not the known estrogen target genes *Greb1* and *Pgr*. Ctrl+IgG n = 4; E2B+IgG n = 4; Ctrl+Ly6G n = 3; E2B + Ly6G n = 3. White and black bars represent Ctrl and E2B treatment, respectively. Statistical significance was evaluated by unpaired two-tailed Student’s t-tests; *p < 0.05, **p < 0.01, ****p < 0.0001.

Since neutrophils in both the peripheral blood and tissues express functionally active ERα and ERβ^22^,^23^, we studied if estrogen targets neutrophils specifically to induce the inflammatory gene signatures. Mammary neutrophils were isolated using biotin-labelled Gr-1 antibody(RB6-8C5)^24^. FACS analysis of the unbound cell population confirmed that Ly6G^hi^ neutrophils, but not Ly6G^int^ monocytes, were depleted(Supplementary Fig. 3a,b). Real-time PCR analysis demonstrated that all 10 inflammatory genes tested(*S100a8, S100a9, Ccl6, Ccl9, Cxcl1, Cxcl2, Clec4d, Clec4e, Saa3* and *Cox-2*) were significantly up-regulated by E2B in isolated neutrophils(Fig. 4a).

To validate that estrogen induces the expression of the immune response genes in neutrophils, we determined the effect of neutrophil depletion on estrogen-induced gene expression in mammary tissue using Ly6G antibody clone 1A8. Depletion efficacy in the peripheral blood and mammary tissue was 95% and 90%, respectively(Fig. 4b). Neutrophil depletion did not affect E2B-induced expression of *bona fide* estrogen target genes *Pgr* and *Greb1*, nor did it affect E2B-induced down-regulation of *Rb1* gene(Fig. 4c). On the other hand, neutrophil depletion abolished E2B-induced expression of inflammatory genes *S100a8, S100a9, Ccl6, Ccl9, Cxcl2, Clec4d* and *Clec4e* in the mammary gland(Fig. 4d). Neutrophil depletion also abolished E2B-induced expression of tissue remodeling genes *Vcan, Mmp3, Mmp9* and *Timp1*. It also appears that estrogen-induced expression of *Cox2* in mammary gland occurs in neutrophils because it is abolished upon neutrophil depletion. These observations substantiate the notion that estrogen exerts distinct gene regulatory effect in the neutrophils of the mammary tissue.

### Estrogen promotes tumor initiation in postpartum mammary gland through neutrophil recruitment

**Figure 5:**
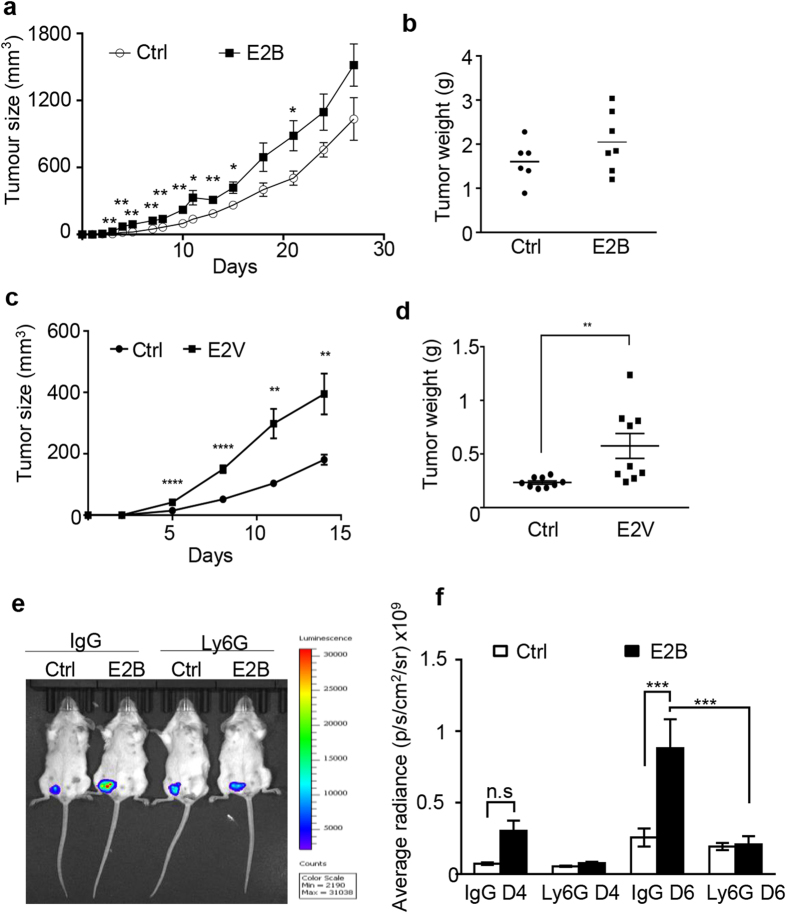
Estrogen-stimulated neutrophils are required for estrogen-induced mammary tumor growth in involuting mammary tissue. (**a**–**d**) Estrogen stimulated markedly ERα-negative 4T1 cell growth in involuting mammary gland.(**a**,**b**) 0.5 × 10^6^ 4T1-luc2 cells were injected into the ninth abdominal mammary gland at 24 h post-weaning. The mice were treated with Ctrl(n = 7) or E2B(n = 8) in sesame oil daily for the first 7 days after tumor injection. Volumes of palpable tumors were measured by digital calipers at 2–3 days intervals(**a**). Tumor weight at end point(day 37)(**b**). Ctrl n = 6;E2B n = 8.(**c**,**d**) Involuting mice were implanted with Ctrl or estradiol valerate(E2V) pellets at 24 h post-weaning. 1 × 10^6^ 4T1-luc2 cells were injected into the ninth abdominal mammary gland 24 h after the hormone pellet implantation. Volumes of palpable tumors were measured by digital calipers at 3 days intervals(**c**). Tumor weights at the time of sacrifice(day 15)(**d**). Ctrl n = 9; E2V n = 9.(**e**,**f**) Neutrophil depletion abolished E2B-induced tumor growth. Mice at 24 h post-weaning were given either isotype control IgG or Ly6G antibody followed by Ctrl or E2B treatment in sesame oil and were injected with 1 × 10^6^ 4T1-luc2 cells. Bioluminescence imaging of tumors was performed on day 4 and day 6 of tumor inoculation. Ctrl+IgG n = 4, E2B+IgG n = 4, Ctrl+Ly6G n = 4, E2B+Ly6G n = 4. Representative bioluminescence image of tumor-bearing mouse from each of the four groups on day 6 of tumor injection(**e**). Bioluminescent quantification of tumor growth(**f**). For figures(**a**–**d**), statistical significance was evaluated by unpaired two-tailed Student’s t-tests; for figure(**f**), statistical significance was evaluated by one way ANOVA followed by post-hoc Tukey’s multiple comparisons test, **p < 0.01, ***p < 0.001.

Studies have suggested that inflammation during mammary involution provides a favourable environment for mammary malignant growth^3^,^4^. We asked if estrogen-induced inflammatory responses in mammary gland during involution serve as an additional drive for mammary tumor growth. Syngeneic 4T1-BALB/c tumor model was used to address this question. 4T1 is an ERα-negative cell line derived from a mammary tumor of MMTV + BALB/c mouse and form spontaneous tumor in BALB/c mice^25^. This model enables the evaluation of the influence of estrogen on tumor development through tissue microenvironment in the context of non-compromised immunity. In the first experiment(5a and 5b), mice at 24 h involution received daily injections of E2B or control vehicle for the first 7 days following tumor cell inoculation. Estrogen treatment significantly accelerated 4T1 mammary tumor progression during the 7 days of estrogen treatment as compared to vehicle-treated controls(Fig. 5a). This effect lingered on for another 1 week after the last dose of E2B treatment. Thereafter, there were no significant differences in tumor sizes and tumor weight at the time of harvest between estrogen and vehicle-treated groups(Fig. 5b), confirming that the presence of estrogen drove the growth of these ERα-negative mammary tumors. In the second experiment(Fig. 5c,d), mice were implanted with a 90-day slow-release estrogen pellet(25 mg/pellet) or a placebo pellet on the first day of weaning. tumor cells were inoculated into the 9th mammary gland the next day and the tumors were harvested two weeks later. Figure 5c shows that estrogen treatment markedly increased tumor size in the involuting mammary gland at all time-points measured. The significance of the difference between E2B-treated and control group is greater(p < 0.0001) at the early time points(day 5 and 8) than that at the later time points(day 12 and 14)(p < 0.01). It is possible that the effect of E2B is boasted by the inflammatory condition in the mammary tissue at the earlier time points. With continuous release of estrogen from the pellet, the tumor weight at the time of harvest is significantly higher(p < 0.01) in E2B-treated group than the control(Fig. 5d). These data confirm that estrogen exposure during involution accelerates tumor development by influencing the tissue environment.

Since estrogen markedly enhanced the recruitment of neutrophils and promoted neutrophil expression of protumoral cytokines and chemokines, we evaluated the involvement of neutrophils in estrogen-stimulated tumor growth. Mice were injected with 4T1-luc2 cells at 24 h post-weaning. Control IgG or Ly6G-specific antibody 1A8 was injected daily for 5 days starting from 24 h post-weaning to deplete neutrophils. Bioluminescent imaging showed that E2B treatment significantly promoted tumor growth after 4 and 6 days of tumor inoculation in control IgG-treated mice(Fig. 5e,f). Ly6G antibody abolished E2B-stimulated tumor initiation and growth. This indicates that estrogen-elicited neutrophil infiltration and reprogramming play a key role in estrogen-induced tumor growth in the involuting mammary gland.

## Discussion

Estrogen is well-known to promote mammary ductal growth. It has also been shown to induce macrophage and eosinophil infiltration that are required for postnatal mammary development^26^,^27^. This study reveals a hitherto unrecognized feature of estrogen action that heightens mammary inflammation and promotes neutrophil infiltration. This is associated with estrogen-induced growth of ERα-negative mammary tumors. More importantly, systemic neutrophil depletion abolishes estrogen-induced mammary tumor growth in mice undergoing mammary involution. The observations suggest that estrogen reprograms neutrophils to create a protumoral microenvironment in involuting mammary tissue. Indeed, estrogen induces neutrophil expression of a plethora of genes encoding protumoral cytokines/chemokines(e.g. CXCL1, CXCL2, S100A8, S100A9), matrix metalloproteinases(MMP3, MMP9) and COX-2 that are all known to promote tumor growth and metastasis^28^. For example, over-expression of CXCL1 and CXCL2 favors cancer cell survival in metastatic sites through the induction of S100A8 and S100A9 secretion from CD11b+Gr1+cells^29^. MMPs are well-known modulators of tumor microenvironment and regulate pathways that control growth, inflammation, and angiogenesis^28^. COX-2 is also widely known to promote breast cancer progression and metastasis^30^. The combined effects of estrogen on the protumoral microenvironment via neutrophils, and on the induction of growth promoting genes(e.g. *Greb1, Areg, Pgr*) and inhibition of tumor repressor gene *Rb1* in mammary epithelial cells would conceivably facilitate the malignant progression of preneoplastic lesions of the breast. We propose therefore that estrogen exposure during post-weaning mammary involution can significantly increase the risk of developing PABC.

To our knowledge, this is the first report of estrogen-induced neutrophil infiltration in mammary gland. Macrophages have been reported to stimulate neutrophil recruitment to the site of inflammation through the secretion of chemokines such as CXCL1 and CXCL2^31^,^32^,^33^. Macrophages were also found to be essential for lipopolysaccharides-induced neutrophil recruitment in the mammary gland^34^. However, E2B did not induce macrophage infiltration or increase the gene expression of neutrophil chemoattractants such as CXCL1, CXCL2 and S100A9 in isolated F4/80+ macrophages(Data not shown). Macrophages are therefore unlikely involved in E2B-induced neutrophils recruitment during mammary involution. On the other hand, estrogen-induced neutrophil infiltration is associated with upregulation in neutrophils of *S100a8, S100a9, Cxcl1, Cxcl2, Ccl6* and *Ccl9* that are well-known neutrophil chemoattractants encoding genes^35^. Since there is already significant neutrophil infiltration at the time of estrogen treatment(24 h post-weaning)^16^, neutrophil-derived cytokines and chemokines are likely responsible for estrogen-induced mammary neutrophil infiltration during involution. This notion is also consistent with the understanding that the initial small number of neutrophils at the inflammatory site is able to trigger additional neutrophil infiltration^36^.

It is also interesting that the effect of estrogen on neutrophil infiltration and the expression of inflammatory genes in mammary neutrophils is specific to the mammary gland undergoing involution. This distinct response of the involuting mammary gland to estrogen suggests a plastic nature of estrogen action that is dependent on the tissue microenvironment. We postulate that the inflammation during mammary involution modifies the epigenetics of the cells in the mammary tissue. This is supported by the report that inflammatory cytokine TNFα can reshape the genomic action of estrogen in breast cancer cells MCF-7 through redistribution of NF-kB and FoxA1 binding across the genome, leading to the remodeling of ERα cistrome^37^. Indeed, TNF activity is heightened during the early phase of involution with 6 members of the TNF superfamily(*Tnf, Tnfsf4, Tnfsf6, Tnfsf7, Tnfsf10* and *Tnfsf12*) induced at 12 h post weaning^38^. It is plausible that the heightened activity of TNF/NF-kB during mammary involution caused a global shift of ER cistrome and hence distinct response to estrogen. Furthermore, 4 of the top 5 upstream regulators(IKBKB, TNFSF12, IKBKG and CHUK/IKK1)(Supplementary Table 3) of estrogen regulated genes in our study are linked to NF-kB activation^39^. This could elicit a positive feedback loop to further heighten and sustain NF-kB-induced remodeling of ERα cistrome. It should be noted that the mammary tissue contains several cell types including mammary epithelial cells and neutrophils that can be subjected to epigenetic modifications in response to inflammation. Future studies will verify inflammation-induced global shift in ER cistrome of different cell types.

Our study suggests that estrogen exposure during mammary involution may increase the risk of parity-associated breast cancer. The E2B dose at 20 μg/kg is physiologically relevant based on the understanding that E2B at 50 μg/kg can give rise to estradiol concentrations of 40–120 pg/ml at peak level^40^,^41^, and the estradiol levels in a non-ovariectomised mice fluctuates from 20 to 70 pg/ml depending on the stages of the estrus cycle^42^. Mice resume their estrus cycle within 2–5 days after weaning^43^. The significance of intra-mammary estrogen has been demonstrated by the report that overexpression of aromatase in the involuted mammary gland of mice resulted in the development of 70–80% ductal hyperplasia^44^. Contraceptive pills or environmental estrogens may also be significant sources of exogenous estrogen.

In summary, this study reveals a new dimension of estrogen action that is fundamentally shaped by the mammary environment during involution. Estrogen heightens mammary inflammation during involution by eliciting neutrophil infiltration. More importantly, the study established that estrogen elicits a fundamental reprogramming of neutrophil activity to build a tissue microenvironment favorable for tumor development. This effect together with its known function of inhibiting tumor suppressor genes and stimulating growth-promoting genes would conceivably promote mammary malignant progression. Furthermore, estrogen-induced expression of *Cox-2* in neutrophils could, in turn, increase the expression of estrogen synthesizing enzyme, aromatase, through its main product prostaglandin E2^45^, initiating a positive feedback loop to further increase local production of estrogen and aggravate the tumor-promoting effect. Thus, inhibition of estrogen action during post-weaning mammary involution would be a useful strategy for the prevention of PABC. This unique effect of estrogen during mammary involution suggests a malleable nature of estrogen action that may be exploited for modifying estrogen signaling in breast cancer treatment.

## Materials and Methods

### Cell culture

Mouse mammary gland adenocarcinoma tumor cell line 4T1-luc2 was obtained from Caliper(Mountain View, CA). Cells were maintained in RPMI 1640 medium(GE Healthcare) containing 10% fetal bovine serum(Sigma-Aldrich) and 2 mM L-glutamine(GE healthcare). Cells were incubated at 37 °C in a humidified atmosphere of 5% CO_2_-95% air. 4T1-luc2 was verified to be free of mycoplasma contamination.

### Mice studies

Animals used in this study complied with all relevant federal guidelines(Institutional Animal Care and Use Committee(IACUC Protocol No: ARF SBS/NIE-A009)), and was approved by Nanyang Technological University and the National Advisory Committee for Laboratory Animal Research(NACLAR), Singapore.

#### Ovariectomy and estrogen treatment

Female BALB/c mice were mated at 6–8 weeks of age. Bilateral ovariectomy was performed on lactating mice at day 2 post-partum. Age-matched nulliparous control were also ovariectomized at the same time to remove the source of endogenous estrogens. The pup size was standardized at 5–6 to each lactating mouse. Involution was initiated by forced weaning on lactating day 12–14. 17β-estradiol-3-benzoate(E2B)(Sigma-Aldrich) dissolved in benzyl alcohol was administrated subcutaneously at 20 μg/kg of body weight per day in sesame oil. Control(Ctrl) animals received sesame oil only. The mice were randomly assigned to Ctrl or E2B groups. To collect tissue and blood for analysis, the animals were sacrificed under anaesthesia by cervical dislocation. Mammary tissues were collected and snap frozen in liquid nitrogen and stored at −80 °C until analysis. For histology, mammary gland tissues were fixed in 4% paraformaldehyde(PFA)(Sigma-Aldrich) for 24–48 h before transferring to 70% ethanol for subsequent processing.

#### Effect of estrogen on tumor growth during mammary involution

For tumor growth study, mice were treated with vehicle control or E2B for 7 days or implanted subcutaneously with placebo or estradiol valerate pellets(E2V, 2.5 mg, 90 days release, Innovative research of America) starting at 24 h post-weaning. At 48 h post-weaning, 0.5 or one million 4T1-luc2 cells were injected into the ninth abdominal mammary gland. Tumor volume was determined after the tumor became palpable by measuring the external length(L) and width(W) of the tumors using digital calliper. Tumor volume was calculated using the formula V = 0.5 × L × W^2^, where L and W represent the largest tumor diameter and perpendicular tumor diameter, respectively.

#### *In vivo* neutrophil depletion

Neutrophil depletion procedures were adapted from a previous study^46^. Briefly, each mouse was administered i.p. with 20 μg of monoclonal rat anti-Ly6G antibody(1A8 clone, BioXCell) or control IgG(clone 2A3, BioXCell). The first antibody injection was given at 24 h post-weaning 6 h before the first E2B injection. The mice were then received a second dose of 20 μg of antibody and E2B on the next day before they were harvested at 72 h post-weaning.

To evaluate the effect of neutrophil depletion on tumor growth study, each mouse was administered i.p. with anti-Ly6G or control IgG antibodies. The first dose of antibody(20 μg/mouse) was given at 6 h before the injection of tumor cells(4T1-luc2) and E2B injection at 24 h post-weaning. Each mouse then received a daily dose of 10 ug antibody and hormone treatment for 5 days. Neutrophil depletion efficiency was measured by FACS analysis of mammary and blood samples.

To evaluate the effect of neutrophil depletion on estrogen-induced growth of 4T1-luc2 tumor, luciferase substrate D-luciferin(Caliper Life Sciences) was given to the mice intraperitoneally(i.p.) at a dose of 150 mg/kg. Mice were anaesthetized by 2.5%(vol/vol) gaseous isoflurane in oxygen and placed beneath the cool charge coupled device camera in an IVIS Lumina II imaging system(Caliper Life Sciences). Ventral images of the mice were acquired at 5 minutes after D-luciferin injections using LivingImage 4.3. The area injected with 4T1-luc2 cell was selected as the region of interest(ROI) on bioluminescent images. The average radiance(p/s/cm2/sr) from that ROI was used as the measurement for analysis.

### Histological analysis

PFA-fixed mammary tissue samples were paraffin-embedded by the Institute of Molecular and Cell Biology Core Histopathology Laboratory, The Advanced Molecular Pathology Laboratory(AMPL). Paraffin-embedded mammary tissue samples(4^th^ inguinal mammary gland) were sectioned at 5 μm. Immunohistochemical(IHC) staining was carried out using the VECTASTAIN^®^ Elite_®_ ABC Kit(Vector laboratories) and biotinylated-Gr1 antibody(clone RB6-8C5)(eBiosciences)(1:100). The tissue sections were counterstained with Richard-Allan Scientific^TM^ Signature Series Hematoxylin 2 and scanned using Axio Scan.Z1. Neutrophils were identified and counted based on the criteria of a positive IHC stain and a discernible banded or segmented nucleus. To ensure uniformity in area selection, an area of 20 mm^2^ around the lymph node was demarcated using the ZEN 2 Lite software. Neutrophils within blood vessels were excluded in the counting.

### Tissue RNA isolation and Real Time PCR(RT-PCR)

Mammary gland samples were lysed in Trizol reagent(Life technologies) for isolation of total RNA. After extraction, RNA was digested with RNase-Free DNase(Promega), followed by clean up with RNeasy mini kit(Qiagen). Reverse transcription was carried out using random primers(Promega) and SuperScript II™ reverse transcriptase(Life Technologies) according to manufacturer’s instructions. Briefly, RNA was incubated with 0.25 μg random primers and heat-denatured at 70 °C for 10 mins followed by immediate cooling on ice for 3 mins. The reaction was then incubated with a mixture containing 5X first strand buffer, 0.01 mM dithiothreitol(DTT), and 0.4 mM dNTPs for 10 mins at 25 °C, then 42 °C for 2 mins before incubating with reverse transcriptase for 50 mins. To inactivate the reverse transcriptase, the reaction was heated for another 10 mins at 72 °C.

RT-PCR was carried out with SYBR Green master mix(KAPA Biosystems) on an ABI Prism 7500 Sequence Detection System or Quantstudio 6 Flex RT- PCR System(Applied Biosystems). RT-PCR for each target gene was performed in duplicates. Acidic ribosomal phosphoprotein P0(36B4) primers were included in each experiment to normalize the amount of cDNA used. For quantitative analysis, the comparative Threshold Cycle(*C*_t_) method was used, while normalizing to *C*_t_ value of 36B4 in the same sample. The data is expressed as relative fold changes in arbitrary values. Primers are listed in Supplementary Table 1.

### Microarray analysis using the GeneChip Mouse Gene 2.0 ST array

Total RNA was isolated from mammary tissues and hybridized onto Mouse Gene 2.0 ST Array(Affymetrix) using the GeneChip WT PLUS Reagent Kit(Affymetrix) at the OrigeneLabs(Research Instruments, Singapore) following the manufacturer’s protocol. After hybridization, the array chips were stained and washed using an Affymetrix Fluidics Station 450/250 and finally scanned using the Affymetrix^®^ Gene Chip Scanner 3000. The scanned images were inspected for hybridization efficiency and.CEL files generated from Gene Chip Command Console Software(AGCC) were imported into Expression Console(EC) software v.1.2 for array quality control(QC). For each sample, the probe values were summarized using robust multi-array average(RMA) algorithm embedded in the EC software, which comprises of convolution background correction, quantile normalization, and median polish summarization.

The data analysis was carried out using the Partek^®^ Genomics Suite™ 6.0(Partek Inc., St. Louis, MO). Normalized data for all samples were first analyzed by hierarchical cluster analysis(HCA) using heat maps to find genes cluster based on similarity in their expression pattern. Default parameters were used where the expression of each gene was standardized to mean 0 and standard deviation of 1. Genes, which were unchanged, were displayed as a value of zero and coloured black. Up-regulated genes had positive values and displayed as green. Down-regulated genes had negative values and were displayed as red. Statistically significant differentially expressed genes(DEGs) between E2B and Ctrl involuting mammary glands were selected using one-way analysis of variance(ANOVA). The final list of DEGs was selected based on fold change(FC) ≥ 1.5 with a p- value < 0.05.

### Gene Ontology and Pathway Analysis

Genes whose expression were significantly regulated(fold-change >1.5; p value < 0.05) were subjected to gene ontology(GO) and pathway analysis. The gene ontology(GO) enrichment was determined using a chi-square test comparing the proportion of the transcript list in an ontology, to the proportion of the background gene list in that same ontology. Functional groups with >5 genes and an enrichment score >3 were considered as significant. Different GO terms and pathway was determined using the Partek Genomic Suite 6.0 and GeneGo Metacore analysis platforms. Ingenuity Pathway Analysis IPA Suite was also used to obtain upstream regulators of estrogen-induced genes.

### Mammary gland digestion and cell isolation

The mammary gland tissues(removed of the draining lymph nodes) were minced and then digested for 1 h at 37 °C with gentle shaking in 1 mg/mL collagenase(Sigma) and 40U DNAse(Sigma) containing Dulbecco’s Modified Eagle’s Medium(DMEM). The digested samples were then filtered through a 100 μm nylon sieve to obtain single cell suspension. The subsequent mixture was centrifuged at 450rcf for 5 mins, before removing the supernatant and re-suspending the pellet in 1 mL of erythrocyte lysis buffer to lyse erythrocytes. 9 ml of DMEM media was added to inactivate the lysis reaction. The cells were pelleted and re-suspended in 1 mL of DMEM. The number of viable cells was counted in Trypan Blue using a haemocytometer. One million cells from each sample were used for the FACS staining reaction. Circulating leucocytes were obtained from heparinized blood extracted by cardiac puncture. Residual erythrocytes were removed by hypotonic lysis in erythrocyte lysis buffer. Leucocytes, neutrophils or macrophages were isolated from the mammary gland digests by positive selection with the Dynabeads^®^ Biotin binder kit(Invitrogen) coupled with biotinylated-antibodies directed against the respective cell specific surface markers. Separation efficacy was determined by flow cytometry. Resulting cell mixtures deprived of leucocytes, neutrophils or macrophages were also kept as controls for subsequent analysis.

### Flow cytometry

Flow cytometry studies of single cell suspension from blood and mammary tissue were performed on LSRII(BD Biosciences), and subsequently analyzed with FlowJo software(Tree Star). The following fluorochrome-conjugated mAbs purchased from either eBioscience or Biolegend were used: CD45(clone 30F11), Gr-1(clone RB6-8C5), Ly6G(clone 1A8), CD11b(clone M1/70). Dead cells were identified with fixable viability dye(eBioscience).

### Statistics

Outlier samples were excluded based on Grubbs’ test^47^. Data were expressed as means ± SEM. Statistical significance of the RT-PCR, FACS and histological counts data between two treatments were determined by unpaired two-tailed Student’s t-test. Equal variance was assumed for all data set for all statistical tests. For tumor studies, statistical significances of the data were analyzed using one way ANOVA followed by post-hoc Tukey. For all tests, difference was considered significant if the p value is less than 0.05.

## Additional Information

**How to cite this article:** Chung, H. H. *et al*. Estrogen reprograms the activity of neutrophils to foster protumoral microenvironment during mammary involution. *Sci. Rep.* **7**, 46485; doi: 10.1038/srep46485(2017).

**Publisher's note:** Springer Nature remains neutral with regard to jurisdictional claims in published maps and institutional affiliations.

## Supplementary Material

Supplementary Information
