# Supplementary material for: Estrogen reprograms the activity of neutrophils to foster protumoral microenvironment during mammary involution
Source: Sci Rep. 2017 Apr 21;7:46485. doi: 10.1038/srep46485 (PMC5399373; doi:10.1038/srep46485)
Supplement: Supplementary Information [file srep46485-s1.pdf]

Estrogen reprograms the activity of neutrophils to foster protumoral microenvironment during mammary involution

[‡]Hwa Hwa Chung, [‡]Yu Zuan Or, Smeeta Shrestha, Jia Tong Loh , Chew Leng Lim, Zoe Ong, Amanda Rui En Woo, I-Hsin Su, *Valerie C-L Lin

[‡]Both authors contributed equally.
School of Biological Sciences, Nanyang Technological University,
60 Nanyang Drive,
Singapore 637551, Singapore

Supplementary Material

Supplementary Table 1.

Gene	Forward (5'-3')	Reverse (5'-3')
36B4	GATCGGGTACCCAACTGTTGCC	CAGGGGCAGCAGCCGCAAATGC
Areg	CACAGCGAGGATGACAAGGA	GAGGATGATGGCAGAGACAAAGA
Ccl6	GCTGGCCTCATACAAGAAATG	AAGCAGCAGTCTGAAGAAGTG
Ccl9	CCCTCTCCTTCCTCATTCTTACA	AGTCTTGAAAGCCCATGTGAAA
Cd44	AGAAAAATGGCCGCTACAGTATC	TGCATGTTTCAAAACCCTTGC
Clec4d	CCGAGAGGAGCCACAGCC	TCATGCCAGGTCTGGTTGTCA
Clec4e	TGCTACAGTGAGGCATCAGG	GGTTTTGTGCGAAAAAGGAA
Cox-2	CCAGCACTTCACCCATCAGTT	ACCCAGGTCCTCGCTTATGA
Cts B	TCCTTGATCCTTCTTTCTTGCC	ACAGTGCCACACAGCTTCTTC
Cxcl1	ATCCAGAGCTTGAAGGTGTTG	GTCTGTCTTCTTTCTCCGTTACTT
Cxcl2	CCAACCACCAGGCTACAG	GCGTCACACTCAAGCTCTG
Greb1	CGGGCTTTGTGAGGAGTCA	CCGGAAGCACAAAGAAGACAGA
Mmp3	TGGCCATCTCTTCCATCCAA	CCCAGAACTGATTTCCTTTAAAAATG
Mmp9	CTGCATTTCTTAAGGACGG	AAGTCGAATCTCCAGACACG
Pgr	CGCCATCTACCAGCCGCTC	TGAATCCGGCCTCAGGTAGTT
Rb1	TGCATCTTTATCGCAGCAGTT	GTTCACACGTCCGTTCTAATTTG
S100a8	CGAAAACTTGTTCAGAGAATTGGA	ACTTTTATCACCATCGCAAGGAA
S100a9	GTTGATCTTTGCCTGTCATGAG	AGCCATTCCCTTTAGACTTGG
Saa3	GCAGCACGAGCAGGATGAAGCC	GCTCGCCACATGTCTCTAGACCC
Timp1	GATATGCCCACAAGTCCCAGAACC	GCACACCCCACAGCCAGCACTAT
Tnfα	TGAACTTCGGGGTGATCGGTC	AGCCTTGTCCCTTGAAGAGAAC
Vcan	TCCTGATTGGCATTAGTGAAG	CTGGTCTCCGCTGTATCC
Vegf	ATGGATGTCTACCAGCGAAG	CTGAACAAGGCTCACAGTGA

Supplementary Table 1. List of RT- PCR primers used in the study.

Supplementary Table 2.

SN	Gene Symbol	Gene Name	p-value (treat vs. control)	Fold-Change (treat vs. control)
1	*S100a8*	S100 calcium binding protein A8 (calgranulin A)	0.006	9.244
2	*Stfa2l1*	stefin A2 like 1	0.003	7.003
3	*S100a9*	S100 calcium binding protein A9 (calgranulin B)	0.004	4.517
4	*Cxcl2*	chemokine (C-X-C motif) ligand 2	0.012	4.509
5	*Stfa1*	stefin A1	0.004	4.253
6	*Slc5a8*	solute carrier family 5 (iodide transporter), member 8	0.015	3.422
7	*Clec4d*	C-type lectin domain family 4, member d	0.001	3.376
8	*Clec4e*	C-type lectin domain family 4, member e	0.004	3.328
9	*Tnfrsf23*	tumor necrosis factor receptor superfamily, member 23	0.015	3.273
10	*Saa3*	serum amyloid A 3	0.024	3.040
11	*Anxa2*	annexin A2	0.001	2.999
12	*Ecm1*	extracellular matrix protein 1	0.012	2.786
13	*Gm14636*	predicted gene 14636	0.022	2.767
14	*Ccl6*	chemokine (C-C motif) ligand 6	0.003	2.745
15	*Ptx3*	pentraxin related gene	0.012	2.732
16	*Slc7a11*	solute carrier family 7 (cationic amino acid transporter, y+ sy	0.013	2.670
17	*Hp*	haptoglobin	0.005	2.552
18	*Pdcd4*	programmed cell death 4	0.000	2.416
19	*Ifitm6*	interferon induced transmembrane protein 6	0.014	2.408
20	*Ccl9*	chemokine (C-C motif) ligand 9	0.005	2.392
21	*Cxcl1*	chemokine (C-X-C motif) ligand 1	0.009	2.358
22	*Sectm1b*	secreted and transmembrane 1B	0.001	2.337
23	*Rny1*	RNA, Y1 small cytoplasmic, Ro-associated	0.030	2.321
24	*Emp1*	epithelial membrane protein 1	0.024	2.310
25	*Tiparp*	TCDD-inducible poly(ADP-ribose) polymerase	0.007	2.278
26	*Vcan*	versican	0.032	2.256
27	*Tm4sf1*	transmembrane 4 superfamily member 1	0.000	2.231
28	*Slc28a3*	solute carrier family 28 (sodium-coupled nucleoside transporter	0.009	2.229
29	*Ifi205*	interferon activated gene 205	0.001	2.201
30	*Cxcl5*	chemokine (C-X-C motif) ligand 5	0.015	2.197
31	*S100a10*	S100 calcium binding protein A10 (calpactin)	0.001	2.189
32	*Anxa1*	annexin A1	0.014	2.178
33	*Timp1*	tissue inhibitor of metalloproteinase 1	0.009	2.167
34	*Qsox1*	quiescin Q6 sulfhydryl oxidase 1	0.002	2.162
35	*Mir5099*	microRNA 5099	0.009	2.156
36	*Slc7a5*	solute carrier family 7 (cationic amino acid transporter, y+ sys	0.008	2.103
37	*Il1b*	interleukin 1 beta	0.045	2.093
38	*Ifi202b*	interferon activated gene 202B	0.020	2.071
39	*Snord34*	small nucleolar RNA, C	0.009	2.071
40	*Pigr*	polymeric immunoglobulin receptor	0.011	2.042
41	*Slfn4*	schlafen 4	0.005	2.005
42	*LOC100862198*	uncharacterized LOC100862198	0.024	2.002
43	*Ptgs2*	prostaglandin-endoperoxide synthase 2	0.012	1.992
44	*Hspa1b*	heat shock protein 1B	0.029	1.989
45	*Mfap5*	microfibrillar associated protein 5	0.024	1.974
46	*Ppan*	peter pan homolog (Drosophila)	0.034	1.962
47	*Ctsb*	cathepsin B	0.003	1.960
48	*Msr1*	macrophage scavenger receptor 1	0.033	1.944
49	*Tubb6*	tubulin, beta 6 class V	0.047	1.941
50	*Ccl7*	chemokine (C-C motif) ligand 7	0.038	1.928

Supplementary Table 2.

SN	Gene Symbol	Gene Name	p-value (treat vs. control)	Fold-Change (treat vs. control)
51	*Anxa3*	annexin A3	0.006	1.910
52	*Fpr2*	formyl peptide receptor 2	0.013	1.901
53	*Srgap3*	SLIT-ROBO Rho GTPase activating protein 3	0.020	1.901
54	*Mt2*	metallothionein 2	0.006	1.891
55	*Ifi204*	interferon activated gene 204	0.031	1.885
56	*Cd44*	CD44 antigen	0.012	1.877
57	*Ttpa*	tocopherol (alpha) transfer protein	0.008	1.876
58	*Lyve1*	lymphatic vessel endothelial hyaluronan receptor 1	0.032	1.864
59	*Wdr1*	WD repeat domain 1	0.006	1.844
60	*Tfg*	Trk-fused gene	0.017	1.807
61	*S100a4*	S100 calcium binding protein A4	0.027	1.784
62	*Vamp8*	vesicle-associated membrane protein 8	0.006	1.774
63	*2610203C22Rik*	RIKEN cDNA 2610203C22 gene	0.000	1.765
64	*9430008C03Rik*	RIKEN cDNA 9430008C03 gene	0.031	1.757
65	*Klf6*	Kruppel-like factor 6	0.009	1.749
66	*Cblb*	Casitas B-lineage lymphoma b	0.046	1.747
67	*2410042D21Rik*	RIKEN cDNA 2410042D21 gene	0.012	1.744
68	*Acot9*	acyl-CoA thioesterase 9	0.027	1.738
69	*Gm5150*	predicted gene 5150	0.048	1.738
70	*Atf6*	activating transcription factor 6	0.032	1.737
71	*LOC100505155*	uncharacterized LOC100505155	0.049	1.728
72	*Mrc1*	mannose receptor, C type 1	0.006	1.722
73	*Irg1*	immunoresponsive gene 1	0.006	1.720
74	*Tuba1c*	tubulin, alpha 1C	0.005	1.712
75	*Ttc39b*	tetratricopeptide repeat domain 39B	0.012	1.709
76	*Mrgpra2a*	MAS-related GPR, member A2A	0.050	1.705
77	*Zfp410*	zinc finger protein 410	0.003	1.695
78	*Fndc3b*	fibronectin type III domain containing 3B	0.039	1.687
79	*Gm9386*	predicted pseudogene 9386	0.014	1.681
80	*Abhd5*	abhydrolase domain containing 5	0.049	1.678
81	*Mir5125*	microRNA 5125	0.049	1.677
82	*Igsf6*	immunoglobulin superfamily, member 6	0.025	1.669
83	*Plp2*	proteolipid protein 2	0.039	1.668
84	*Ly96*	lymphocyte antigen 96	0.012	1.661
85	*Rny3*	RNA, Y3 small cytoplasmic (associated with Ro protein)	0.010	1.654
86	*Gm19333*	predicted gene, 19333	0.002	1.651
87	*1110017F19Rik*	RIKEN cDNA 1110017F19 gene	0.035	1.649
88	*Cstb*	cystatin B	0.016	1.644
89	*Rpl31*	ribosomal protein L31	0.024	1.644
90	*Prdx3*	peroxiredoxin 3	0.004	1.640
91	*Slc15a4*	solute carrier family 15, member 4	0.015	1.633
92	*Vmn1r103*	vomeronasal 1 receptor 103	0.011	1.627
93	*Tubb2a*	tubulin, beta 2A class IIA	0.022	1.624
94	*Tagln2*	transgelin 2	0.026	1.624
95	*Krt19*	keratin 19	0.040	1.615
96	*Erlin2*	ER lipid raft associated 2	0.035	1.610
97	*Taf5l*	TAF5-like RNA polymerase II, p300	0.004	1.609
98	*Gm19425*	predicted gene, 19425	0.008	1.607
99	*Ssr3*	signal sequence receptor, gamma	0.046	1.606
100	*Csnk1g1*	casein kinase 1, gamma 1	0.027	1.598

Supplementary Table 2.

SN	Gene Symbol	Gene Name	p-value (treat vs. control)	Fold-Change (treat vs. control)
101	*Zc3h7a*	zinc finger CCCH type containing 7 A	0.007	1.596
102	*Ccr1*	chemokine (C-C motif) receptor 1	0.018	1.594
103	*Tnc*	tenascin C	0.044	1.594
104	*Taf9b*	TAF9B RNA polymerase II, TATA box binding protein (TBP)-associ	0.028	1.592
105	*Mrpl10*	mitochondrial ribosomal protein L10	0.001	1.584
106	*Slc25a24*	solute carrier family 25 (mitochondrial carrier, phos	0.033	1.583
107	*Sc4mol*	sterol-C4-methyl oxidase-like	0.017	1.580
108	*Sec61g*	SEC61, gamma subunit	0.022	1.576
109	*H2-Q10*	histocompatibility 2, Q region locus 10	0.048	1.576
110	*Sh3bp2*	SH3-domain binding protein 2	0.029	1.571
111	*Cmas*	cytidine monophospho-N-acetylneuraminic acid synthetase	0.045	1.570
112	*Tomm5*	translocase of outer mitochondrial membrane 5 homolog (yeast)	0.028	1.570
113	*Mt1*	metallothionein 1	0.000	1.569
114	*Timm9*	translocase of inner mitochondrial membrane 9 homolog (yeast)	0.016	1.568
115	*Mir3109*	microRNA 3109	0.001	1.564
116	*Lypd5*	Ly6	0.016	1.564
117	*Spred1*	sprouty protein with EVH-1 domain 1, related sequence	0.034	1.560
118	*F630028O10Rik*	RIKEN cDNA F630028O10 gene	0.023	1.560
119	*Trim27*	tripartite motif-containing 27	0.004	1.559
120	*D1Ertd622e*	DNA segment, Chr 1, ERATO Doi 622, expressed	0.029	1.558
121	*Purb*	purine rich element binding protein B	0.019	1.555
122	*Wsb1*	WD repeat and SOCS box-containing 1	0.050	1.552
123	*Naa25*	N(alpha)-acetyltransferase 25, NatB auxiliary subunit	0.006	1.549
124	*Plp2*	proteolipid protein 2	0.010	1.548
125	*Gm4184*	predicted gene 4184	0.016	1.548
126	*5830416P10Rik*	RIKEN cDNA 5830416P10 gene	0.014	1.547
127	*Ctdsp1*	CTD (carboxy-terminal domain, RNA polymerase II, polype	0.028	1.547
128	*Fosl2*	fos-like antigen 2	0.015	1.537
129	*Nt5dc3*	5'-nucleotidase domain containing 3	0.004	1.536
130	*Hspa2*	heat shock protein 2	0.010	1.535
131	*Pcyt1a*	phosphate cytidylyltransferase 1, choline, alpha isoform	0.027	1.535
132	*Nme1*	NME	0.010	1.533
133	*Chka*	choline kinase alpha	0.016	1.533
134	*Tuba1c*	tubulin, alpha 1C	0.005	1.526
135	*Ssr1*	signal sequence receptor, alpha	0.038	1.526
136	*Cfl2*	cofilin 2, muscle	0.033	1.523
137	*Tbcd*	tubulin-specific chaperone d	0.010	1.520
138	*Ildr2*	immunoglobulin-like domain containing receptor 2	0.010	1.519
139	*I830127L07Rik*	RIKEN cDNA I830127L07 gene	0.019	1.519
140	*Snord89*	small nucleolar RNA, C	0.031	1.519
141	*Opn3*	opsin 3	0.004	1.514
142	*Ssr1*	signal sequence receptor, alpha	0.010	1.511
143	*Morf4l2*	mortality factor 4 like 2	0.014	1.507
144	*Nme2*	NME	0.027	1.506
145	*Gm4701*	predicted gene 4701	0.037	1.506

Supplementary Table 2.

SN	Gene Symbol	Gene Name	p-value (treat vs. control)	Fold-Change (treat vs. control)
146	*Hbegf*	heparin-binding EGF-like growth factor	0.048	1.501
147	*Vegfa*	vascular endothelial growth factor A	0.037	-1.502
148	*Cib2*	calcium and integrin binding family member 2	0.009	-1.504
149	*Olfr510*	olfactory receptor 510	0.012	-1.508
150	*Jrkl*	jerky homolog-like (mouse)	0.035	-1.509
151	*C030019I05Rik*	RIKEN cDNA C030019I05 gene	0.044	-1.509
152	*Neurog2*	neurogenin 2	0.036	-1.511
153	*Mir1928*	microRNA 1928	0.011	-1.511
154	*Olfr917*	olfactory receptor 917	0.043	-1.512
155	*Zfp125*	zinc finger protein 125	0.046	-1.512
156	*Cited2*	Cbp	0.037	-1.515
157	*Gamt*	guanidinoacetate methyltransferase	0.003	-1.520
158	*1700010M22Rik*	RIKEN cDNA 1700010M22 gene	0.046	-1.520
159	*Insig1*	insulin induced gene 1	0.047	-1.523
160	*Il15*	interleukin 15	0.010	-1.525
161	*Arfrp1*	ADP-ribosylation factor related protein 1	0.042	-1.528
162	*Syne1*	synaptic nuclear envelope 1	0.048	-1.531
163	*Ccdc34*	coiled-coil domain containing 34	0.029	-1.532
164	*Odz4*	odd Oz	0.028	-1.535
165	*Rdh14*	retinol dehydrogenase 14 (all-trans and 9-cis)	0.020	-1.535
166	*Dcbld1*	discoidin, CUB and LCCL domain containing 1	0.038	-1.539
167	*Cypt3*	cysteine-rich perinuclear theca 3	0.029	-1.545
168	*Plscr5*	phospholipid scramblase family, member 5	0.031	-1.546
169	*G6b*	G6B protein	0.027	-1.549
170	*Snora21*	small nucleolar RNA, H	0.013	-1.549
171	*Hscb*	HscB iron-sulfur cluster co-chaperone homolog (E. coli)	0.030	-1.553
172	*Gm6260*	predicted gene 6260	0.006	-1.554
173	*Foxb2*	forkhead box B2	0.036	-1.554
174	*Gm16675*	predicted gene, 16675	0.033	-1.556
175	*Trim43c*	tripartite motif-containing 43C	0.034	-1.559
176	*Prx*	periaxin	0.012	-1.560
177	*Yy2*	Yy2 transcription factor	0.021	-1.560
178	*Adam4*	a disintegrin and metallopeptidase domain 4	0.026	-1.563
179	*Qrfpr*	pyroglutamylated RFamide peptide receptor	0.006	-1.565
180	*Gsdma*	gasdermin A	0.018	-1.568
181	*Gm20033*	predicted gene, 20033	0.029	-1.575
182	*Pla2g15*	phospholipase A2, group XV	0.023	-1.575
183	*Fxyd3*	FXYD domain-containing ion transport regulator 3	0.029	-1.581
184	*Impa2*	inositol (myo)-1(or 4)-monophosphatase 2	0.015	-1.581
185	*Mkl2*	MKL	0.010	-1.581
186	*Igf1*	insulin-like growth factor 1	0.022	-1.585
187	*Arhgap42*	Rho GTPase activating protein 42	0.042	-1.590
188	*C3*	complement component 3	0.040	-1.591
189	*Col23a1*	collagen, type XXIII, alpha 1	0.005	-1.593
190	*Itgbl1*	integrin, beta-like 1	0.041	-1.594
191	*Zfp119a*	zinc finger protein 119a	0.009	-1.598
192	*Arl13b*	ADP-ribosylation factor-like 13B	0.018	-1.601
193	*Cxcr6*	chemokine (C-X-C motif) receptor 6	0.001	-1.603
194	*Stxbp6*	syntaxin binding protein 6 (amisyn)	0.002	-1.613
195	*Lgr4*	leucine-rich repeat-containing G protein-coupled receptor 4	0.002	-1.620
196	*Taf5*	TAF5 RNA polymerase II, TATA box binding protein (TBP)-associated	0.010	-1.625

Supplementary Table 2.

SN	Gene Symbol	Gene Name	p-value (treat vs. control)	Fold-Change (treat vs. control)
197	*Syne1*	synaptic nuclear envelope 1	0.029	-1.631
198	*Ankrd42*	ankyrin repeat domain 42	0.028	-1.636
199	*Macrod1*	MACRO domain containing 1	0.040	-1.642
200	*Isoc1*	isochorismatase domain containing 1	0.030	-1.645
201	*2210408F21Rik*	RIKEN cDNA 2210408F21 gene	0.006	-1.645
202	*Gm10516*	predicted gene 10516	0.044	-1.656
203	*Igfbp4*	insulin-like growth factor binding protein 4	0.014	-1.666
204	*Tff1*	trefoil factor 1	0.027	-1.673
205	*Mx2*	myxovirus (influenza virus) resistance 2	0.024	-1.678
206	*Rb1*	retinoblastoma 1	0.010	-1.682
207	*Itga8*	integrin alpha 8	0.023	-1.687
208	*D19Ertd737e*	DNA segment, Chr 19, ERATO Doi 737, expressed	0.037	-1.691
209	*4930594M22Rik*	RIKEN cDNA 4930594M22 gene	0.043	-1.695
210	*3110057O12Rik*	RIKEN cDNA 3110057O12 gene	0.048	-1.700
211	*Gramd3*	GRAM domain containing 3	0.031	-1.705
212	*Pot1a*	protection of telomeres 1A	0.041	-1.713
213	*Ndrg1*	N-myc downstream regulated gene 1	0.031	-1.714
214	*Ccdc85c*	coiled-coil domain containing 85C	0.001	-1.740
215	*5830418K08Rik*	RIKEN cDNA 5830418K08 gene	0.035	-1.752
216	*Efcab2*	EF-hand calcium binding domain 2	0.005	-1.761
217	*Il21r*	interleukin 21 receptor	0.034	-1.769
218	*Mir149*	microRNA 149	0.041	-1.782
219	*Gm20290*	predicted gene, 20290	0.029	-1.817
220	*Fads2*	fatty acid desaturase 2	0.029	-1.831
221	*Gm11362*	predicted gene 11362	0.009	-1.849
222	*Csprs*	component of Sp100-rs	0.030	-1.850
223	*Atp5g1*	ATP synthase, H+ transporting, mitochondrial F0 complex	0.025	-1.877
224	*Ces1f*	carboxylesterase 1F	0.030	-1.887
225	*Aym1*	activator of yeast meiotic promoters 1	0.039	-1.893
226	*A930005I04Rik*	RIKEN cDNA A930005I04 gene	0.006	-1.902
227	*Zfp161*	zinc finger protein 161	0.013	-1.934
228	*Gnptab*	N-acetylglucosamine-1-phosphate transferase, alpha and beta s	0.004	-1.974
229	*Gm5088*	poly(A)-binding protein, cytoplasmic pseudogene	0.025	-1.986
230	*Gm5124*	nucleolar and coiled-body phosphoprotein 1 pseudogene	0.009	-1.990
231	*Zfp764*	zinc finger protein 764	0.045	-2.018
232	*D530049I02Rik*	RIKEN cDNA D530049I02 gene	0.045	-2.053
233	*Lrrcc1*	leucine rich repeat and coiled-coil domain containing 1	0.039	-2.081
234	*Gm14391*	predicted gene 14391	0.037	-2.146
235	*Gbp11*	guanylate binding protein 11	0.014	-2.272
236	*Acsl1*	acyl-CoA synthetase long-chain family member 1	0.010	-2.428
237	*Fam13a*	family with sequence similarity 13, member A	0.020	-2.497
238	*Mir680-2*	microRNA 680-2	0.005	-2.759
239	*Arhgef6*	Rac	0.010	-2.980

Supplementary Table 2. List of 239 genes with fold change greater than 1.5 and are significantly regulated ($p<0.05$) in mammary glands of mice at 48h post-weaning treated with E2B for 24h as compared to the Ctrl. Gene expression was profiled using Affymetrix Mouse GeneChip 2.0 ST array.

Supplementary Fig.1

a

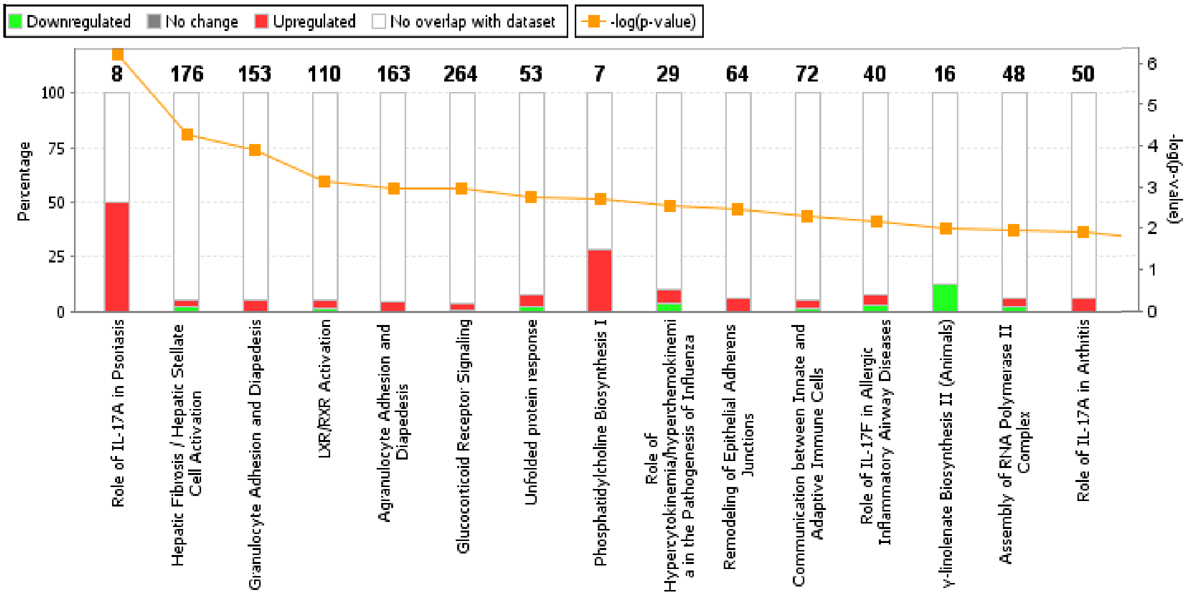

b

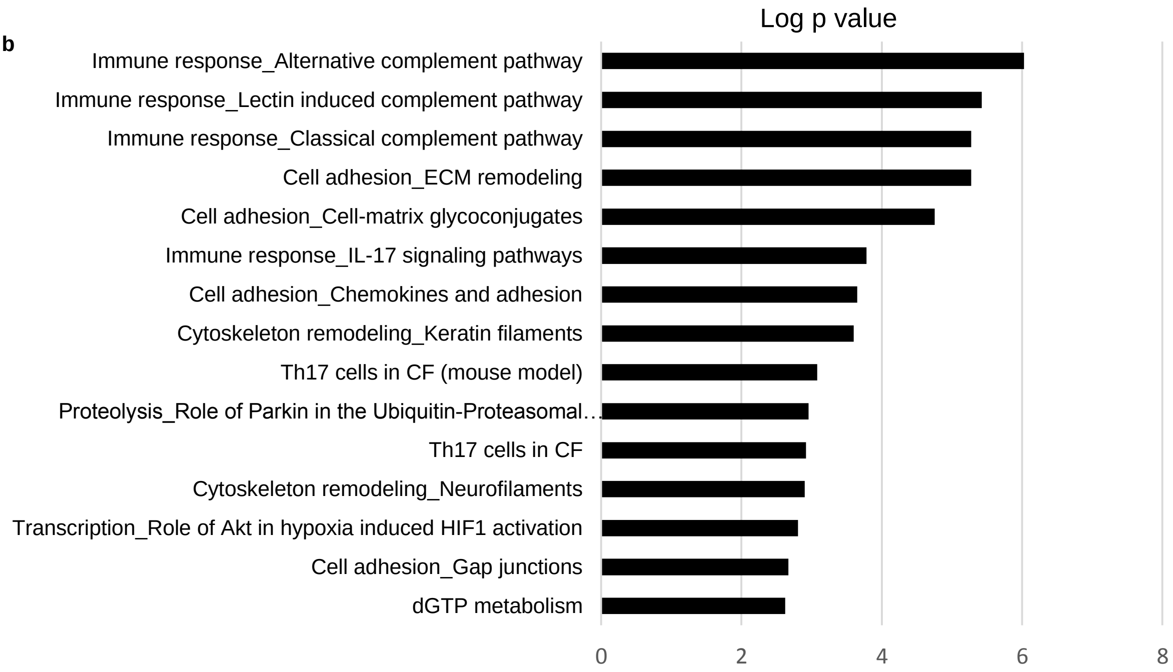

Supplementary Fig.1. Top fifteen canonical pathways that were significantly impacted by estrogen in involuting mammary glands as identified by the two methods of pathway analysis. (a) The Ingenuity Pathway Analysis (IPA) was used to analyse the DEGs with fold change > 1.5 and $p < 0.05$ as listed in the Supplementary Table 2 to obtain major canonical pathways altered in the mammary glands of mice at 48h post weaning treated with Ctrl or E2B in sesame oil for 24h. The stacked bar chart displays the percentage of genes that were upregulated (red), downregulated (green), and genes not overlapping with our data set (white) in each canonical pathway. The numerical value at the top of each bar represents the total number of genes in the canonical pathway. The secondary y-axis (right) shows the −log of p-value calculated by Fisher's exact test p-value, which indicates the significance of each pathway. (b) MetaCore-GeneGo software was used to analyse DEGs listed in Supplementary Table 2. Significant estrogen regulated pathways (listed in y-axis) are displayed against –log (p-value). Hypergeometric intersection was used to estimate p-value, the lower p-value means higher relevance. $p < 0.05$ and False Discovery Rates (FDR) < 0.05 were used as criterion.

Supplementary Table 3.

Upstream Regulator	Predicted activation	Activation Z score	P value of overlap	Target Molecules
IKBKB	Activated	2.462	1.10E-16	*C3,Ccl9,CCR1,CD44,CTSB,CXCL2,CXCL3,CXCL6,HLA-A,IFI16,IL1B,IRG1,Mt1,Mt2,PTGS2,PTX3,S100A4,Saa3,VCAN,VEGFA*
TNFSF12	Activated	3.592	2.37E-14	*Ccl7,Ccl9,CCR1,CLEC4D,CLEC4E,CXCL2,CXCL3,CXCL6,IL1B,PTX3,S100A8,S100A9,TIMP1*
IFNAR1		0.562	1.08E-13	*Ccl9,CLEC4D,CLEC4E,CXCL2,CXCL3,CXCL6,HLA-A,IFI16,IL15,IL1B,IRG1,Ly6a* (includes others),*Mx1/Mx2,PTGS2,SLFN12L*
IKBKG	Activated	2.464	1.43E-13	*C3,Ccl9,CCR1,CTSB,CXCL2,CXCL6,HLA-A,IFI16,IL1B,IRG1,Mt1,Mt2,PTX3,Saa3,VEGFA*
CHUK	Activated	2.464	2.45E-13	*C3,Ccl9,CCR1,CTSB,CXCL2,CXCL3,CXCL6,HLA-A,IFI16,IRG1,Mt1,Mt2,PTX3,Saa3,VEGFA*

Supplementary Table 3. Predicted activation state of top five upstream transcriptional regulators in 24h E2B treated mammary gland at 48h involution**.** IKBKB: Inhibitor Of Kappa Light Polypeptide Gene Enhancer In B-Cells, Kinase Beta, TNFSF12: Tumor Necrosis Factor (Ligand) Superfamily, Member 12, IFNAR1: Interferon (Alpha, Beta And Omega) Receptor 1, IKBKG: Inhibitor Of Kappa Light Polypeptide Gene Enhancer In B-Cells, Kinase Gamma, CHUK: Conserved Helix-Loop-Helix Ubiquitous Kinase. Shown are those genes predicted to be activated based on upstream regulator analysis (z- score) and the p-value of overlap was used to rank the significance of the overlap between the genes targeted by the upstream regulator in the Ingenuity Pathways Knowledge Base (IPKB) database and the experimental dataset.

Supplementary Fig.2

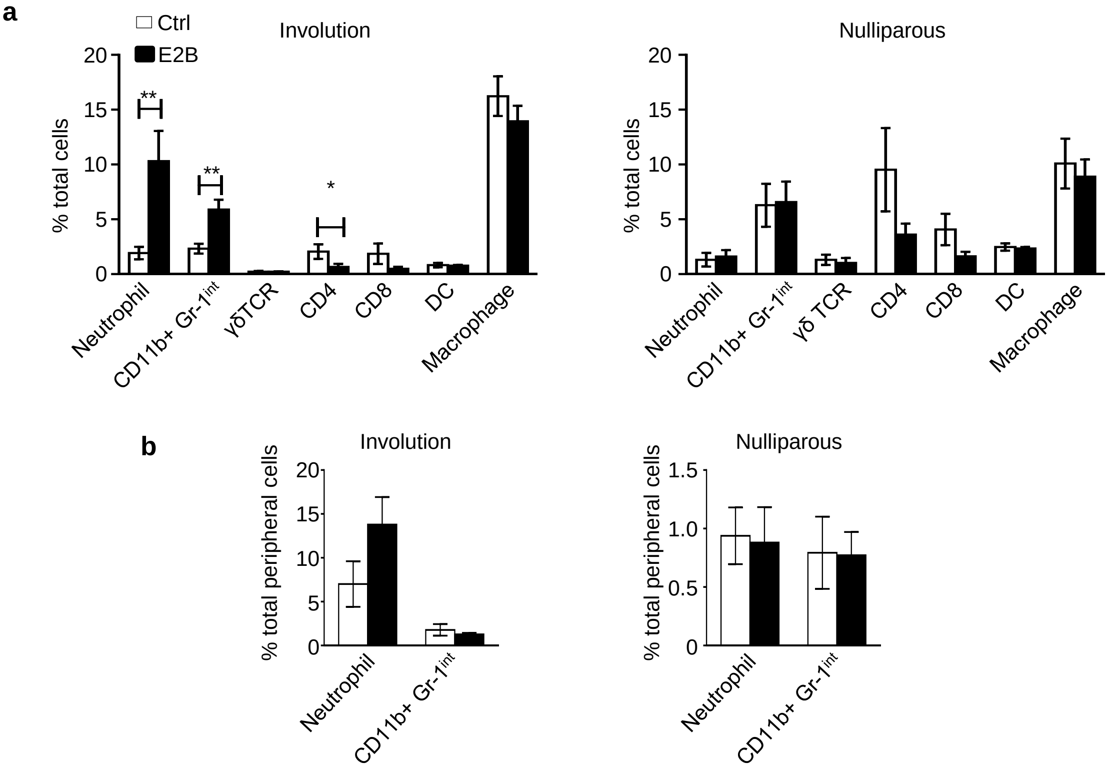

Supplementary Fig.2. (a) Flow cytometric analysis of leucocytes infiltration in mammary glands in response to estrogen treatment in nulliparous and involuting mice. Mammary tissues were digested by collagenase to obtain single cell suspension. Leucocytes derived from mammary glands of nulliparous or involuting mice administered with either Ctrl or E2B for 48h at 24h post-weaning were stained with different surface markers. Neutrophils ($CD45^{+}$ $CD11b^{+}$ Gr-1^{hi}), myeloid-derived monocytic cells ($CD45^{+}$, $CD11b^{+}$ Gr-1^{int}), $CD4^{+}$ T cells ($CD45^{+}CD3^{+}$ $CD4^{+}$), $CD8^{+}$ T cells ($CD45^{+}$ $CD3^{+}$ $CD8^{+}$), γδ T cells ($CD45^{+}$ TCR γδ $^{+}$ $CD3^{+}$), dendritic cells (DC) (CD45+ $MHCII^{+}$ $F4/80^{-}$ $CD11c^{+}$), macrophages (CD45+ $MHCII^{+}$, $F4/80^{+}$). Antibodies purchased from either eBioscience or Biolegend were used: CD45 (clone 30F11), CD3ε (clone 145-2C11), CD4 (clone GK1.5), CD8a (clone 53-6.7), F4/80 (clone CI:A3-1) and TCR γ/δ (clone GL3). For neutrophils and myeloid-derived monocytic cells in mammary tissue from mice undergoing involution, Ctrl n=13, E2B n=13; for macrophages, DC, CD4, CD8, and γδ T cells in involuting mammary glands, Ctrl n=12, E2B n=12. For neutrophils and myeloid-derived monocytic cells in mammary tissue from nulliparous mice, Ctrl n=10, E2B n=9; for mammary macrophages, CD4, CD8 and γδ T cells in nulliparous mice, Ctrl n=6, E2B n=6; for DC in nulliparous mammary glands, Ctrl n=4, E2B n=4. (b) Peripheral blood from nulliparous and involuting mice were collected and stained for neutrophils and myeloid-derived monocytic cells markers. For the data of peripheral neutrophils in nulliparous mice, Ctrl n=8, E2B n=7; for peripheral myeloid-derived monocytic cells in nulliparous mice, Ctrl n=4, E2B n=4; for peripheral neutrophils in involution mice, Ctrl n=10, E2B n=9; for peripheral myeloid-derived monocytic cells in involution mice, Ctrl n=9, E2B n=9. Data was represented as Mean ± SEM. Statistical significance was evaluated by unpaired two-tailed Student's t-tests, $^{**}p<0.01$

Supplementary Fig.3

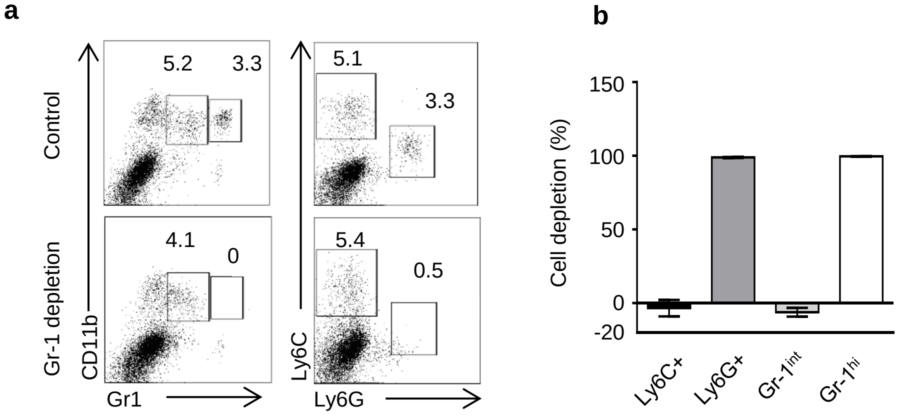

Supplementary Fig.3. (a) Depletion of Ly6G+ but not Ly6C+ cells by Gr-1 antibody was verified by flow cytometry. Mammary tissues from 72h post-weaning were digested and neutrophils were isolated using biotinylated Gr-1 antibody bound to Dynabeads® Streptavidin. Cells that were not bound to the beads were analyzed by flow cytometry. Gr-1 -bound beads collected greater than 95% Ly6G^{hi} neutrophils but did not retain Ly6C+positive or Ly6G^{int} cells. (a) Representative of FACS plot. (b) Depletion efficiency of cells expressing Gr-1+, Ly6G^{hi}, Ly6G^{int} or Ly6C^{+}. Data was represented as Mean ± SEM. Statistical significance was evaluated by unpaired two-tailed Student's t-tests.
